# Vigorous achalasia: Zebra amongst horses

**DOI:** 10.4102/sajr.v24i1.1953

**Published:** 2020-11-30

**Authors:** Jayaranjeetham Jayabalan, Nithin Theckumparampil, Aravintho Natarajan, Dilip S. Phansalkar, George Kurian

**Affiliations:** 1 Department of Radiodiagnosis, Pondicherry Institute of Medical Sciences, Puducherry, India; 2 Department of Gastroenterology, Pondicherry Institute of Medical Sciences, Puducherry, India

**Keywords:** vigorous achalasia, diffuse oesophageal spasm, classic achalasia, barium swallow, proton pump inhibitors

## Abstract

Vigorous achalasia is an oesophageal disorder with clinical and radiological characteristics of classic achalasia and diffuse oesophageal spasm. It is a rarely reported variant. A 60-year-old gentleman presented with complaints of difficulty in swallowing, regurgitation and chest pain for the past 10 years. His symptoms persisted despite the use of proton pump inhibitors. On endoscopy and barium swallow, the diagnosis of vigorous achalasia was confirmed. It is a rare variant of classic achalasia usually misdiagnosed as diffuse oesophageal spasm.

## Introduction

Classic achalasia and diffuse oesophageal spasm (DES) both have different clinical, radiological and manometric features.^1^ Chest pain is more common in DES, whereas it is uncommon in classic achalasia. Regurgitation and retention are more common in classic achalasia, whereas they are uncommon in DES.^2^ At barium swallow imaging, classic achalasia shows dilatation and DES demonstrates a corkscrew appearance. Motility studies indicate that DES and classic achalasia are at opposite ends of a spectrum.^3^ Diffuse oesophageal spasm responds less to treatment, unlike classic achalasia.^4^ Some individuals have a combination of the above-mentioned symptoms and cannot be placed under already established entities.^2^

In 1957, Olsen et al. first described the term vigorous achalasia in a group of individuals with classic achalasia whose clinical features and manometric features demonstrated common features of both classic achalasia and DES.^5^ According to Millan et al.,^3^ vigorous achalasia falls in-between the two extremes (DES and classic achalasia) of the oesophageal motility disorder spectrum.^3^ This case illustrates the unusual oesophageal disorder of vigorous achalasia.

## Case description

A 60-year-old male with no known comorbidities, presented with a 10-year history of difficulty in swallowing (liquids more than solids) and chest pain, which persisted despite the use of proton pump inhibitors. His physical examination and laboratory parameters were unremarkable. A barium swallow study was recommended, followed by upper gastro-intestinal (UGI) endoscopy.

**FIGURE 1:**
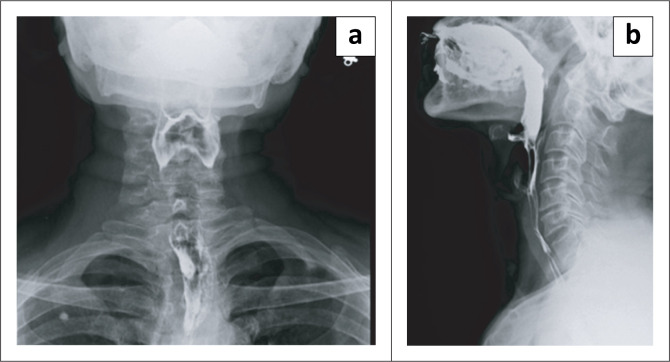
(a, b) Barium swallow Antero -posterior (AP) view and lateral view, barium passed down the cervical and upper thoracic oesophagus smoothly and normally.

**FIGURE 2:**
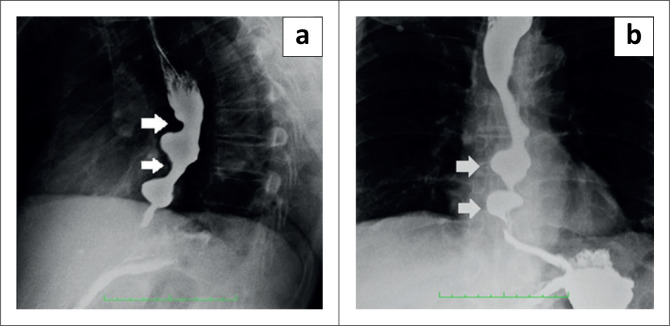
(a) Barium swallow Left anterior oblique (LAO) view – Arrows denote the areas of narrowing in the lower thoracic oesophagus. (b) Barium swallow Antero – posterior (AP) view – Arrows denote the areas of dilatation of the oesophagus proximal to the areas of narrowing.

**FIGURE 3:**
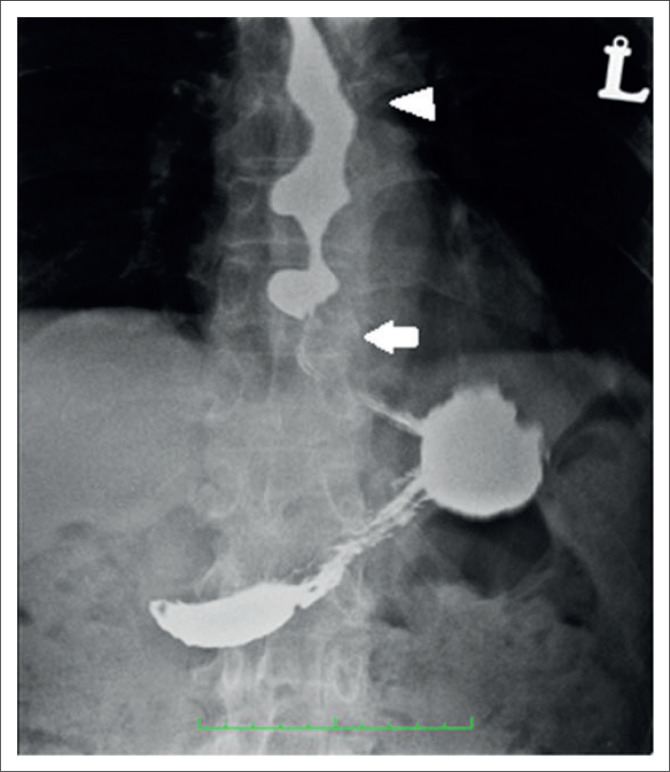
Barium swallow Antero – posterior (AP) view – Arrow denotes the spindle-shaped tapering of the lower end of the esophagus. Arrowhead denotes the tertiary contractions.

During the barium swallow study (using Baritop powder 100% w/v 250 mL), the cervical and proximal part of the thoracic oesophagus showed normal passage of barium (Figure 1). The distal part of the thoracic oesophagus exhibited a few areas of narrowing and areas of dilatation proximal to the narrowed segments (Figure 2). Tertiary contractions were noted and the distal end of the esophagus displayed spindle-shaped tapering (Figure 3).

**FIGURE 4:**
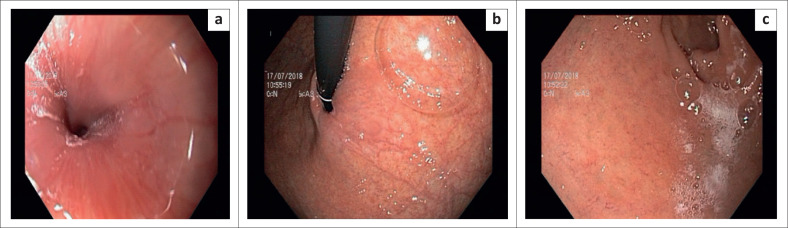
(a) Upper gastro-intestinal endoscopy – Shows the dilated proximal esophagus and stenotic lower oesophageal sphincter (b) Difficulty in passing the endoscope beyond the stenosis (c) Pooling of saliva above the stenotic lower oesophageal sphincter.

At UGI-endoscopy, (Olympus scope – size 2.8 mm), the upper part of the oesophagus was mildly dilated but otherwise normal. The distal part revealed lower oesophageal sphincter (LES) stenosis with pooling of saliva proximal to it. There was difficulty in passing the UGI scope beyond the site of the stenosis (Figure 4).

## Discussion

Vigorous achalasia is an unusual oesophageal disorder that has clinical and radiological characteristics of both achalasia and DES.^5^ According to one study, vigorous achalasia falls in-between the two extremes (DES and classic achalasia) of the oesophageal motility disorder spectrum.^3^ Although manometry is the gold standard test for motility disorders, it is time consuming and not easily available.^6,7^ In this case the radiological findings were very clear.

### Classic achalasia versus vigorous achalasia

Achalasia is an uncommon disorder that involves loss of inhibitory ganglions in the myenteric plexus of the LES and the body of the oesophagus. Over time, the progressive loss of cholinergic neurons results in dilation and low amplitude simultaneous contractions in the oesophageal body, resulting in classic achalasia.^8^

Regurgitation and retention are the most common symptoms in classic achalasia, whereas chest pain is uncommon.^2^ At barium swallow imaging there will be proximal dilatation of the oesophagus with tapering (‘bird beaking’) of the lower end of the oesophagus. Classic achalasia is characterised by the complete absence of peristalsis.^9^ The diameter of the oesophageal body in vigorous achalasia is generally less than that of classic achalasia. As in our case of vigorous achalasia, there is minimal oesophageal dilatation with tertiary contractions and spindle-shaped tapering of the lower end of the oesophagus^10^ (Figure 3).

Endoscopy of classic achalasia shows a dilated tortuous oesophagus with retention of fluid or food.^11^ The manometry study by Goldenberg et al. revealed that the amplitude is less than 37 mm Hg in achalasia, whereas it is greater than or equal to 37 mm Hg in patients with vigorous achalasia.^12^ Previous studies on the management of these conditions revealed that vigorous achalasia is less responsive to pneumatic dilatation when compared with achalasia and have recommended surgery.^2,13^ In a study by Okike et al.,^14^ the thoracic approach of an extended oesophageal myotomy of the oesophagus to the point where there are excess pressures and/or repetitive waves, was found to be better than pneumatic dilatation. Later, a study by Paricio et al.^15^ showed that Heller’s myotomy, not extended to the oesophageal body, via an abdominal approach, also yields better results in vigorous achalasia as compared with classic achalasia.^15^ Peroral endoscopic myotomy (POEM) is a minimally invasive endoscopic treatment for symptomatic oesophageal achalasia.^16^ It was found to be effective in nearly 93.7% of patients with type III or vigorous achalasia.^17^

### Diffuse oesophageal spasm versus vigorous achalasia

Diffuse oesophageal spasm (DES) is a rare motility disorder characterised by chest pain, dysphagia and intermittent increased simultaneous contractions.^18^ Impairment of the oesophageal inhibitory pathway results in premature contractions in the distal muscularis propria. Thus, the pathophysiology is similar to achalasia.^19^ Chest pain is the most common symptom in DES, whereas it is uncommon in vigorous achalasia.^2^ In our case, the patient experienced both dysphagia and chest pain. Physiologically, the studies on motility revealed that DES is the opposite of achalasia.^3^ Diffuse oesophageal spasm responds less to treatment, unlike achalasia.^4^

Endoscopy can sometimes show simultaneous ring-shaped contractions.^20^ At barium swallow imaging, DES shows interruption of the normal peristalsis by multiple, repetitive, non-propulsive oesophageal contractions (tertiary peristaltic waves) resulting in the characteristic corkscrew oesophagus.^21^ Manometric criteria includes the presence of simultaneous contractions in the distal (smooth muscle) oesophagus in more than 10% of wet swallows with an amplitude contraction of at least 30 mm Hg, alternating with normal peristalsis.^22^ Comparatively, vigorous achalasia is an atypical variant with non-peristaltic contractions giving the appearance of a corkscrew oesophagus on barium swallow^23^ (Figure 3).

The clinical features, imaging findings, endoscopic and manometric findings of oesophageal motility disorders are summarised in Table 1.

**TABLE 1:** Characteristic features of the oesophageal motility disorders.

Characteristics	Classic achalasia	Diffuse Oesophageal spasm	Vigorous achalasia
Dysphagia	+++	+	++
Regurgitation & retention of food	+++	-	+
Chest pain	+/−	+++	++
**Endoscopy**			
Oesophageal dilatation	+++	-	+
Retention of food	++	-	+/−
Passage of scope	Difficulty (depending on grade of stenosis)	Easy passage of scope	With difficulty can be passed through
**Barium swallow**			
Proximal & mid part	Dilated and tortuous	Corkscrew pattern (Multiple simultaneous contractions with intervening normal peristalsis)	Mildly dilated with few tertiary contractions
Distal part	Bird beaking	Corkscrew pattern	Spindle-shaped
Manometry amplitude	< 37 mm Hg.	30 mm Hg	≥ 37 mm Hg

## Conclusion

Vigorous achalasia is a rare oesophageal motility disorder, commonly misdiagnosed as DES. Knowledge and recognition of this uncommon entity will help in early recognition and optimal management.
